# Reduced suppressive effect of β_2_-adrenoceptor agonist on fibrocyte function in severe asthma

**DOI:** 10.1186/s12931-017-0678-7

**Published:** 2017-11-21

**Authors:** Chun-Yu Lo, Charalambos Michaeloudes, Pankaj K. Bhavsar, Chien-Da Huang, Po-Jui Chang, Chun-Hua Wang, Han-Pin Kuo, Kian Fan Chung

**Affiliations:** 1 Airway Disease Section, National Heart and Lung Institute, Imperial College London and Biomedical Research Unit, Royal Brompton Hospital, London, UK; 2 Department of Thoracic Medicine, Chang Gung Medical Foundation, Chang Gung University College of Medicine, Taipei, Taiwan; 3 Airway Disease, National Heart and Lung Institute, Imperial College London, Dovehouse Street, London, SW3 6LY UK

**Keywords:** Fibrocytes, Severe asthma, β_2_-adrenergic receptor, Corticosteroids, cAMP

## Abstract

**Background:**

Patients with severe asthma have increased airway remodelling and elevated numbers of circulating fibrocytes with enhanced myofibroblastic differentiation capacity, despite being treated with high doses of corticosteroids, and long acting β_2_-adrenergic receptor (AR) agonists (LABAs). We determined the effect of β_2_-AR agonists, alone or in combination with corticosteroids, on fibrocyte function.

**Methods:**

Non-adherent non-T cells from peripheral blood mononuclear cells isolated from healthy subjects and patients with non-severe or severe asthma were treated with the β_2_-AR agonist, salmeterol, in the presence or absence of the corticosteroid dexamethasone. The number of fibrocytes (collagen I^+^/CD45^+^ cells) and differentiating fibrocytes (α-smooth muscle actin^+^ cells), and the expression of CC chemokine receptor 7 and of β_2_-AR were determined using flow cytometry. The role of cyclic adenosine monophosphate (cAMP) was elucidated using the cAMP analogue 8-bromoadenosine 3′,5′-cyclic monophosphate (8-Br-cAMP) and the phosphodiesterase type IV (PDE4) inhibitor, rolipram.

**Results:**

Salmeterol reduced the proliferation, myofibroblastic differentiation and CCR7 expression of fibrocytes from healthy subjects and non-severe asthma patients. Fibrocytes from severe asthma patients had a lower baseline surface β_2_-AR expression and were relatively insensitive to salmeterol but not to 8-Br-cAMP or rolipram. Dexamethasone increased β_2_-AR expression and enhanced the inhibitory effect of salmeterol on severe asthma fibrocyte differentiation.

**Conclusions:**

Fibrocytes from patients with severe asthma are relatively insensitive to the inhibitory effects of salmeterol, an effect which is reversed by combination with corticosteroids.

**Electronic supplementary material:**

The online version of this article (10.1186/s12931-017-0678-7) contains supplementary material, which is available to authorized users.

## Background

Asthmatic inflammation is characterised by extensive airway remodelling involving sub-epithelial fibrosis and thickening of the airway smooth muscle (ASM) layer [1]. 5–10% of asthmatic patients suffer from severe asthma, which is difficult to control despite receiving high-dose inhaled corticosteroids and long-acting β_2_-adrenoceptor (β_2_-AR) agonists (LABA), leukotriene modifiers or theophylline [2]. In severe asthmatic airways, there is more prominent sub-epithelial fibrosis and ASM thickening which contribute to airway obstruction [3].

Fibrocytes are bone marrow-derived progenitor cells that express leukocytes markers, such as CD45, and mesenchymal proteins, including collagen I [4]. Migration of fibrocytes towards allergen-exposed airways and their subsequent differentiation into α–smooth muscle actin (SMA)-expressing myofibroblasts may contribute to increased ASM mass and sub-epithelial fibrosis [5]. Fibrocyte accumulation has also been observed in the airways of severe refractory asthmatic patients [6]. The CC chemokine receptor 7 (CCR7)/CC chemokine ligand 19 axis is important in the migration of fibrocytes from asthmatic patients with chronic airflow obstruction [7]. We have previously shown that severe asthma patients have more circulating fibrocytes, with higher myofibroblastic differentiation potential and relative corticosteroid insensitivity in vitro [8].

The use of LABAs in conjunction with inhaled corticosteroids results in further improvement of symptoms and lung function [9]. β_2_-AR activation induces the production of 3′,5′-cyclic adenosine monophosphate (cAMP) by adenylyl cyclase leading to airway smooth muscle relaxation [10]. β_2_-AR agonists also exert other effects on cellular function such as inhibition of proliferation, α–SMA expression and collagen synthesis in pulmonary fibroblasts [11, 12]. Corticosteroid insensitivity in peripheral blood mononuclear cells (PBMCs) from patients with severe asthma has been associated with reduced glucocorticoid receptor (GR) nuclear translocation and hyper-phosphorylation of GR, effects that are reversed by β_2_-AR agonists [13].

We hypothesised that β_2_-AR activation has differential effects on the function of fibrocytes from severe asthma patients compared to healthy subjects and non-severe asthma patients. We determined the effect of the β2-AR agonist, salmeterol, in the presence or absence of the corticosteroid dexamethasone on the number, differentiation potential, and CCR7 and β_2_-AR expression in fibrocytes from patients with non-severe and severe asthma and healthy subjects. The involvement of cAMP in the downstream signalling was investigated using the phosphodiesterase (PDE) 4 inhibitor rolipram and the cAMP analogue 8-bromoadenosine 3′,5′-cyclic monophosphate (8-Br-cAMP) on fibrocytes in cultured non-adherent non-T (NANT) cells from severe asthmatic subjects.

## Methods

### Subjects

Patients with severe and non-severe asthma and healthy subjects were recruited as previously described [8], according to the American Thoracic Society guidelines for refractory asthma (Table 1) [14]. The study was approved by the local ethics committee and informed consent was obtained from each participant. Additional details on the method are provided in the Additional file 1.

**Table 1:** Clinical characteristics of study subjects

	Healthy	Non-severe Asthma	Severe Asthma
Number	12	9	13
Age, years	39.8 ± 2.5	52.6 ± 4.2	54.4 ± 3.8*
Gender, Female/Male	6/6	7/2	11/2
Inhaled corticosteroid dose, μg BDP equivalent	0	150.0 ± 105.2	2000 ± 258.2**^##^
Atopy (n)	5	4	5
Receiving oral corticosteroids	0	0	4
Pre-bronchodilator FEV_1_ (L)	3.4 ± 0.2	2.2 ± 0.3	1.5 ± 0.2***
Pre-bronchodilator FEV_1_ of predicted value (%)	94.9 ± 2.5	82.3 ± 5.5	61.0 ± 5.5***
FEV_1_/FVC (%)	88.2 ± 2.3	69.5 ± 2.0	64.5 ± 3.1***

Data are expressed as mean ± SEM

*BDP* beclomethasone dipropionate, *FEV1* forced expiratory volume in 1 s, *FVC* forced vital capacity

^*^ *p* < 0.05, ^***^ *p* < 0.001 compared to healthy subjects. ^##^ *p* < 0.01 compared to patients with non-severe asthma

### Isolation of NANT cells

NANT cells were isolated as previously described [8]. Briefly, PBMCs were isolated from peripheral blood using Ficoll-Paque™ PLUS density gradient centrifugation (GE HealthCare, Uppsala, Sweden). Adherent cells were depleted by adherence, and T cells in the non-adherent fraction of PBMCs were removed using CD3^+^ MicroBeads according to the manufacturer’s instructions (Miltenyi Biotec, California, USA). The NANT cells were then incubated in Iscove’s Modified Dulbecco’s Medium (IMDM; Sigma-Aldrich) supplemented with 30% FBS (Sigma-Aldrich) and 1% BSA in a humidified incubator, at 37^∘^C with 5% CO_2,_ for 3 days in the presence or absence of treatments. NANT cells were counted on a haemocytometer after Trypan blue staining.

### Analysis of fibrocytes by flow cytometry

Fibrocytes were identified in the NANT cell population, as cells positive for collagen I and CD45 (Col I^+^/CD45^+^), by flow cytometry [8]. NANT cells were fixed using 0.5% paraformaldehyde, washed twice with phosphate buffered saline (PBS) and incubated with an allophycocyanin (APC)-conjugated mouse anti-human CD45 antibody (BD Biosciences, #555485, San Jose, California, USA). In some experiments cells were also stained with a phycoerythrin (PE)–conjugated mouse anti-human CCR7 antibody (BD Biosciences, #150503) 0.125 μg for 30 min. Cells were then permeabilised using 1X BD FACS Permeabilising Solution 2 (BD Biosciences, #347692) 120 μl for 15 min and then stained using 0.015 μg of fluorescein isothiocyanate (FITC)-conjugated mouse anti-human collagen I antibody (Millipore Corporation, #FCMAB412F, Temecula, California) for 30 min.. Differentiating fibrocytes were identified as α–SMA-positive (α–SMA^+^) cells, by staining permeabilised NANT cells with 0.125 μg of PE-conjugated mouse anti-human α–SMA antibody (R&D Systems, #IC1420P Abingdon, UK). Cells incubated with isotype-matched antibodies were used as negative controls.

To evaluate the expression of β_2_-AR on the surface, cells were incubated with 1 μg of unconjugated rabbit IgG anti-human β_2_-AR (Abcam, #ab61778, Cambridge, UK,) and 0.015 μg of anti-human CD45 antibody for 30 min, followed by a PE–conjugated goat IgG anti-rabbit polyclonal secondary antibody (Abcam, #ab97070) 0.3 μg for 30 min, permeablised and then stained with FITC-conjugated anti-human collagen I antibody 0.5 μg for 30 min.

To evaluate the expression of β_2_-AR in the whole cell, cells were incubated with anti-human CD45 antibody, permeablised, then stained with 1 μg of unconjugated rabbit IgG anti-human β_2_-AR and 0.5 μg of FITC-conjugated anti-human collagen I antibody for 30 min, followed by 0.5 μ of PE–conjugated goat IgG anti-rabbit polyclonal secondary antibody g for 30 min.

### Analysis of NANT cell apoptosis using Annexin V and propidium iodide (PI) staining

NANT cell apoptosis was determined by Annexin V and propidium iodide (PI) staining using the FITC Annexin V/Dead cell apoptosis kit (Invitrogen, Paisley, UK) according to the manufacturer’s instructions. Briefly, following treatment, NANT cells were washed in PBS, incubated with FITC-conjugated annexin V and PI and analysed by flow cytometry. Annexin V^−^/PI^−^ were considered live cells, Annexin V^+^/PI^−^ as early apoptotic and annexin V^+^/PI^+^ as late apoptotic cells.

### Statistical analysis

Data are presented as mean ± standard error of the mean (SEM). Statistical analysis was carried out using the GraphPad Prism v.5 software package (GraphPad Prism Software Inc., California, USA). Intra-group comparisons of more than two conditions were carried out using the Friedman test followed by Dunn’s post-hoc test and pairwise comparisons using a paired t-test. Inter-group comparisons were carried out using the Mann-Whitney test. *p* < 0.05 was considered as statistically significant.

## Results

### Effect of salmeterol on fibrocyte number, differentiation and CCR7 expression

At the time of isolation from blood (day 0) the total number of NANT cells were not significantly different between groups, however there were higher percentages of fibrocytes in severe asthma (51 ± 11 *× 10* ^*3*^; 15% ± 2.2) compared to non-severe asthma (43 ± 5 × *10* ^*3*^; 6.7% ± 1 and healthy subjects (15 ± 4.2 *× 10* ^*3*^; 4.1% ± 0.9). For experiments, 1 × 10^6^ NANT cells per well were seeded at day 0. After three days in culture, there was a concomitant increase in the percentage of fibrocytes within each group (Additional file 2: Figure S3A), suggesting proliferation of fibrocytes in culture. In contrast to day 0, at day 3 there were no significant differences in the percentage of fibrocytes across the groups. However, there was an higher percentage of differentiated (α–SMA+) fibrocytes in the severe asthma group compared to non-severe asthma patients and healthy subjects (Additional file 2: Figure S3B) confirming our previous observations [8]. As the vast majority of α–SMA^+^ cells also expressed the fibrocyte markers Col I and CD45, we chose to identify differentiating fibrocytes by single α–SMA staining (Additional file 3: Figure S1).

**Fig. 1:**
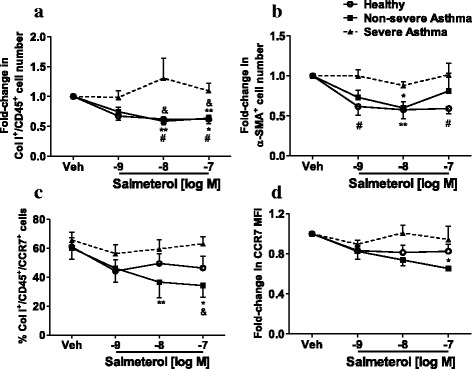
Effect of salmeterol on the number, differentiation and CCR7 expression of fibrocytes. NANT cells from healthy subjects (*n* = 8–9) and patients with non-severe (*n* = 7) or severe asthma (*n* = 7–9) were treated with salmeterol (10^−9^- 10^−7^ M) for 3 days. Fibrocyte (Col I^+^/CD45^+^ cells; **a**) and differentiating fibrocyte (α-SMA^+^ cells; **b**) number, and percentage (**c**) and median fluorescence intensity (MFI; **d**) of CCR7^+^ fibrocytes (Col I^+^/CD45^+^/CCR7^+^ cells) were determined. Data points represent mean ± SEM. * *p* < 0.05 and ** *p* < 0.01 versus vehicle-treated cells for each group. ^#^ *p <* 0.05 healthy versus severe asthma and ^&^ *p <* 0.05 non-severe asthma versus severe asthma

Incubation with salmeterol (10^−9^ - 10^−7^ M) for 3 days led to a decrease in the number of fibrocytes (Fig. 1a *)* and differentiating fibrocytes (Fig. 1b *)* from healthy subjects and non-severe asthma patients. Salmeterol did not modulate the percentage of apoptotic NANT cells (Additional file 4: Figure S2). As we have shown that fibrocytes proliferate in culture the reduction in fibrocyte number by salmeterol is possibly a result of inhibition of proliferation. In contrast, salmeterol did not modulate the number of fibrocytes (Fig. 1a) and differentiating fibrocytes (Fig. 1b) from patients with severe asthma. We also show that there is an increase in the absolute number of fibrocytes in culture at day 3 in all three groups suggesting proliferation of fibrocytes in culture. Pre-treatment with salmeterol inhibited this proliferation in fibrocytes from healthy subjects and non-severe asthmatics but the anti-proliferative effect of salmeterol was not observed in severe asthma. The effect of salmeterol on the absolute numbers of fibrocytes and differentiating fibrocytes is shown in Additional file 2: Figure S3. Salmeterol also led to a decrease in the percentage of CCR7-positive fibrocytes (Fig. 1c) and CCR7 MFI ratio (Fig. 1d) in NANT cells from patients with non-severe asthma but had no significant effect on those from healthy subjects and patients with severe asthma.

The suppressive effect of salmeterol on the number of fibrocytes and differentiating fibrocytes from healthy subjects was prevented in the presence of the β_2_-AR-specific antagonist ICI-118,551 (10^−5^ M; Additional file 5: Figure S4), confirming that the effects of salmeterol are mediated by β_2_-AR activation. The above findings suggest altered β_2_-AR signalling in fibrocytes from severe asthma.

### Effect of cAMP modulation on fibrocyte number and differentiation

**Fig. 2:**
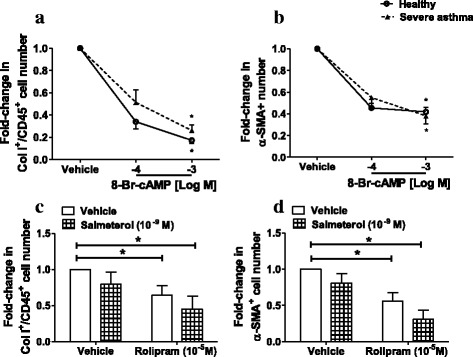
Effect of cAMP modulation on fibrocyte number and differentiation. NANT cells from healthy subjects (*n* = 4) and severe asthma patients (*n* = 4) were treated with 8-Br-cAMP (10^−4^- 10^−3^ M for 3 days (**a**-**b**). Alternatively, NANT cells from severe asthma patients were treated with rolipram (10^−5^ M) in the presence or absence of salmeterol (10^−9^ M) for 3 days (**c**-**d**). The number of fibrocytes (Col I^+^/CD45^+^ cells; **a** and **c**) and differentiating fibrocytes (α-SMA^+^ cells; **b** and **d**) were determined. Data points represent mean ± SEM. * *p* < 0.05 versus vehicle-treated cells for each group

Increasing intracellular cAMP levels using the analogue 8-Br-cAMP (10^−4^ – 10^−3^ M) led to a concentration-dependent decrease in the number of fibrocytes (Fig. 2a) and differentiating fibrocytes (Fig. 2b) from both healthy subjects and severe asthma patients. Also, the PDE4 inhibitor rolipram (10^−5^ M), which inhibits PDE4-dependent cAMP hydrolysis, alone and in combination with salmeterol reduced fibrocyte number and differentiation (Fig. 2c-d). The combination of salmeterol and rolipram had a greater inhibitory effect on the number of fibrocytes and differentiated fibrocytes than either drug alone, although these differences did not reach statistical significance. Together these data suggest that the defect in β_2_-AR signalling in severe asthma fibrocytes possibly lies upstream of cAMP production.

### β_2_-AR expression in fibrocytes from healthy subjects and severe asthma patients

**Fig. 3:**
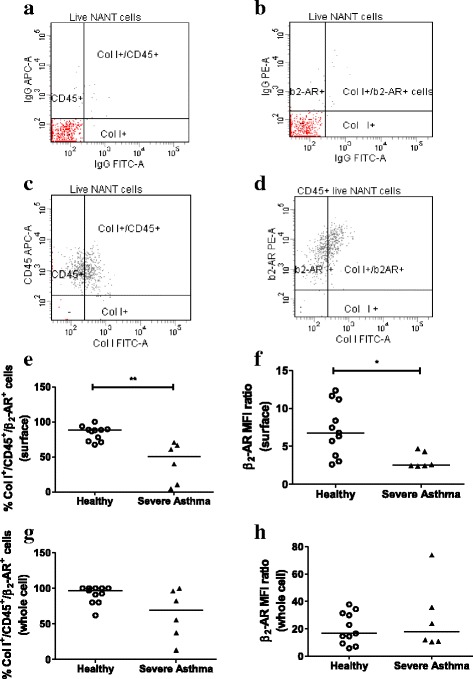
Baseline β_2_-AR expression in fibrocytes from healthy subjects and patients with severe asthma. β_2_-AR expression in fibrocytes from untreated NANT cells of healthy subjects (*n* = 11) and severe asthma patients (*n* = 6) was determined by staining with antibodies for collagen I (Col I), CD45 and β_2_-AR (**c**-**d**), or their respective isotype controls (**a**-**b**) at day 0. Representative flow cytometry scatter plots from one experiment are shown. Surface (**e**-**f**) and whole cell (**g**-**h**) β_2_-AR expression was determined as percentage of β_2_-AR^+^ fibrocytes (Col I^+^/CD45^+^/β_2_-AR^+^ cells; **e, g**) and median fluorescence intensity (MFI) ratios (**f, h**). Horizontal lines represent the median of each group. * *p* < 0.05, ** *p* < 0.01

The baseline, surface and whole cell, expression of β_2_-AR was determined in fibrocytes, in the NANT cells of healthy subjects and severe asthma patients at day 0 by staining with an anti-β_2_-AR antibody before or after permeabilisation, respectively (Fig. 3a-d). Both the percentage of β_2_-AR-positive fibrocytes (Fig. 3e) and the β_2_-AR MFI ratio (Fig. 3f) were lower on the surface of circulating fibrocytes of patients with severe asthma compared to healthy subjects, although whole cell β_2_-AR expression was not significantly different between the two groups (Fig. 3g and h).

**Fig. 4:**
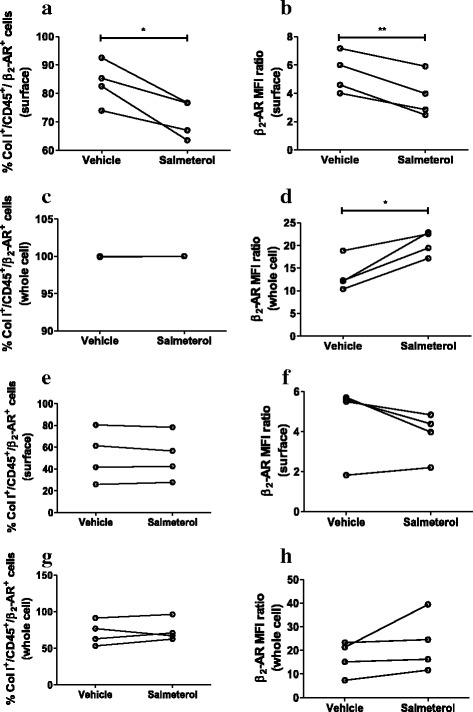
Effect of salmeterol on β_2_-AR expression in fibrocytes from healthy subjects and patients with severe asthma. NANT cells from healthy subjects (**a**-**d**; *n* = 4) and severe asthma patients (**e**-**h**; *n* = 4) were treated with salmeterol (10^−8^ M) for 3 days. Surface (**a**-**b** and **e**-**f**) and whole cell (**c**-**d** and **g**-**h**) β_2_-AR expression was determined as percentage of β_2_-AR^+^ fibrocytes (Col I^+^/CD45^+^/β_2_-AR^+^ cells; **a, c, e, g**) and median fluorescence intensity (MFI) ratios (**b, d, f, h**). * *p* < 0.05 and ** *p* < 0.01

Incubation of NANT cells from healthy subjects with salmeterol (10^−8^ M) for 3 days led to a reduction in the percentage of fibrocytes positive for surface β_2_-AR (Fig. 4a) and the surface β_2_-AR MFI ratio (Fig. 4b). Salmeterol had no effect on the percentage of fibrocytes positive for whole cell β_2_-AR (Fig. 4c) but increased the whole cell MFI ratio (Fig. 4d), in NANT cells from healthy subjects. In contrast, salmeterol had no effect on the percentage of β_2_-AR-positive fibrocytes (ColI^+^/CD45^+^/ β_2_-AR^+^) or the MFI ratio, on the surface (Fig. 4e and f) or in the whole cell (Fig. 4g and h), in NANT cells from patients with severe asthma. These data suggest loss of baseline surface β_2_-AR expression and attenuated agonist-induced β_2_-AR down-regulation in fibrocytes from severe asthma.

### Effect of dexamethasone on salmeterol-induced effects on fibrocyte function

**Fig. 5:**
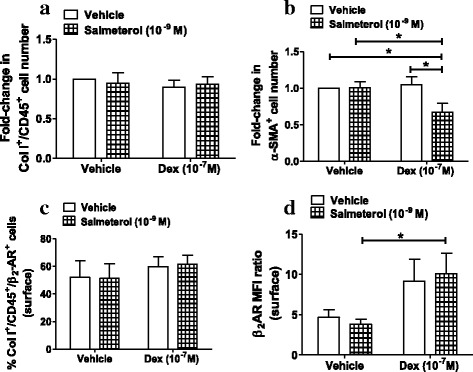
Effect of salmeterol and dexamethasone combination on the number, differentiation and β_2_-AR expression of fibrocytes from severe asthma patients. NANT cells from patients with severe asthma (n = 4–7) were treated with salmeterol (10^−9^ M) in the presence or absence of dexamethasone (Dex; 10^−7^ M) for 3 days. The number of fibrocytes (Col I^+^/CD45^+^ cells; **a**) and differentiating fibrocytes (α-SMA^+^ cells; **b**), as well as the percentage (Col I^+^/CD45^+^/β_2_-AR^+^; **c**) and median fluorescence intensity (MFI) ratio (**d**) of β_2_-AR–positive fibrocytes were determined. Bars represent mean ± SEM. * *p* < 0.05

To investigate whether the addition of a corticosteroid modulates the effects of β_2_-AR activation on fibrocytes of patients with severe asthma, NANT cells were incubated with a submaximal dose of salmeterol (10^−9^ M) in combination with dexamethasone (10^−7^ M). For all groups there was an increase in the total the number fibrocytes at day 3 as described above. Although dexamethasone or salmeterol alone had no effect on fibrocyte number and differentiation, the number of differentiating fibrocytes (Fig. 5b), but not total fibrocytes (Fig. 5a), was reduced by the combination of salmeterol and dexamethasone. Salmeterol and dexamethasone, individually or in combination, had no effect on fibrocytes positive for surface β_2_-AR (Fig. 5c). However, dexamethasone alone led to a non-statistically-significant increase in the β_2_-AR MFI ratio whilst the combination of salmeterol and dexamethasone significantly augmented the β_2_-AR MFI ratio (Fig. 5d). Thus, dexamethasone may potentiate the effects of salmeterol on severe asthma fibrocytes by increasing surface β_2_-AR density.

## Discussion

We have demonstrated that β_2_-AR activation by salmeterol leads to a reduction in the proliferation, myofibroblastic differentiation and CCR7 expression in fibrocytes from healthy subjects and patients with non-severe asthma. In contrast, salmeterol had no effect on fibrocytes from severe asthma patients indicating defective β_2_-AR signalling. Increasing intracellular cAMP levels, using the analogue 8-Br-cAMP or inhibiting cAMP degradation using the PDE4 inhibitor, rolipram, led to reduction in the number and differentiation of severe asthma fibrocytes indicating that the defect possibly lies up-stream of cAMP production. Indeed, we showed that fibrocytes from severe asthma patients have reduced baseline surface β_2_-AR expression which was not reduced further by salmeterol. Co-treatment with dexamethasone increased surface β_2_-AR density and augmented the inhibition of differentiation by salmeterol in fibrocytes of patients with severe asthma.

The airways of patients with severe asthma patients show increased airway remodelling despite receiving high doses of inhaled and oral corticosteroids and LABAs [3]. Increased numbers of circulating fibrocytes, with increased capacity to differentiate into myofibroblasts and relative corticosteroid insensitivity may contribute to the increased airway remodelling in severe asthma [8]. In this study, we show that β_2_-AR activation by salmeterol can reduce the proliferation, differentiation and CCR7 expression of fibrocytes from healthy subjects and non-severe asthmatic patients. These findings are consistent with studies in fibroblasts showing inhibition of collagen synthesis, expression of α–SMA and proliferation in response to β_2_-AR agonists [11, 12, 15].

We also show that fibrocytes from severe asthma are less sensitive to the inhibitory effects of salmeterol suggesting that LABAs may be unable to reduce fibrocyte numbers and myofibroblastic differentiation in the airways of patients with severe asthma due to defective β_2_-AR signalling. Activation of the β_2_-AR leads to G_s_-mediated activation of adenylyl cyclase which catalyses the conversion of adenosine triphosphate (ATP) to cAMP leading to activation of PKA and EPAC-dependent pathways [10]. Increasing intracellular cAMP levels using 8-Br-cAMP mimicked the inhibitory effect of β_2_-AR activation on the number and differentiation of fibrocytes. These findings are in line with studies showing reduction in proliferation, myofibroblastic differentiation and chemotaxis of fibroblasts by cAMP [16–18]. Significantly, fibrocytes from patients with severe asthma were also sensitive to the inhibitory effects of the cAMP analogue, whilst inhibiting cAMP hydrolysis using rolipram enhanced the inhibitory effect of salmeterol on these cells. These findings indicate that the signalling pathways downstream to cAMP are intact in severe asthma fibrocytes and that the defect most likely lies at the level of the β_2_-AR expression or activation.

We have demonstrated that severe asthmatic fibrocytes show reduced surface β_2_-AR expression, whilst they have similar whole cell β_2_-AR expression levels, compared to fibrocytes from healthy subjects. Moreover, salmeterol failed to reduce the surface β_2_-AR expression in fibrocytes from patients with severe asthma, possibly due to the already low β_2_-AR surface density. These findings indicate a possible defect in the membrane localisation but not the overall expression of the β_2_-AR in severe asthma fibrocytes. As this occurs in the absence of salmeterol in vitro, it is possible that this is due to chronic exposure to systemic LABAs or endogenous circulating catecholamines and inflammatory mediators in vivo [19, 20]. Prolonged β_2_-AR activation triggers its uncoupling from adenylyl cyclase, internalisation of the uncoupled receptors, and increased degradation of internalized receptors, leading to desensitisation [21]. Under normal conditions β_2_-AR are subsequently resensitised by recycling back to the cell membrane to interact with agonists [22]. Defective β_2_-AR resensitisation has been shown to cause reduced baseline surface expression and attenuated agonist-induced activation in ASM cells of subjects with fatal asthma [23].

The inhibitory effect of salmeterol on severe asthma fibrocyte differentiation was restored by co-incubation with the corticosteroid, dexamethasone. However, salmeterol and dexamethasone did not modulate the total number of fibrocytes from severe asthma patients in culture and this may be a result of the increased capacity of fibrocytes from patients with severe asthma to survive, as we have previously demonstrated [8]. Resistance to the anti-proliferative effects of corticosteroids, due to reduced expression of CCAAT/enhancer binding protein α (C/EBPα), was also reported in airway smooth muscle cells from patients with asthma [24]. Corticosteroids have been shown to potentiate β_2_-AR agonist effects by increasing β_2_-AR density through up-regulation of transcription [25]. In agreement with these findings, we demonstrated up-regulation of the surface β_2_-AR MFI ratio, indicating increased surface receptor density, in response to dexamethasone and salmeterol in severe asthma fibrocytes. Nonetheless, the superior effect of salmeterol and dexamethasone combination may also be a result of potentiation of glucocorticoid receptor (GR) function by salmeterol, as β_2_-AR agonists are known to induce GR nuclear translocation and modulate phosphorylation of GR, in PBMCs, lung fibroblasts and airway smooth muscle cells [13, 26, 27]. A combination of the corticosteroid, budesonide and the β_2_-AR agonist, formoterol, has been shown to inhibit transforming growth factor (TGF)–β1-induced collagen I synthesis, whilst either drug alone had no effect [28]. Moreover, a salmeterol and fluticasone propionate combination inhibits TGF–β1-induced α-SMA expression in lung fibroblasts [11]. Thus, the combination of β_2_-AR agonists and corticosteroids may be more potent in controlling airway remodelling in severe asthma, than either drug alone.

We showed that rolipram, a first generation selective PDE4 inhibitor [29], enhanced the inhibitory effect of salmeterol on the differentiation of fibrocytes from both healthy subjects and severe asthma patients. Rolipram has been shown to reverse endotoxin-induced airway hyperresponsiveness and bronchoconstriction in vivo [30], whilst PDE4 knockdown attenuates basic fibroblast factor and interleukin-1β-induced proliferation, and transforming growth factor-β-induced myofibroblastic differentiation of fibroblasts [31]. Also, combination of the β_2_-AR agonist, indacaterol, and the PDE4 inhibitor, roflumilast, inhibits the expression of endothelin-1, connective tissue growth factor and α-SMA, and the secretion of fibronectin from normal human lung fibroblasts, more strongly than with either drug alone [32]. Significantly, rolipram was also shown to overcome β_2_-AR desensitisation in ASM cells [33].

## Conclusions

In this study we have demonstrated that fibrocytes from patients with severe asthma show reduced surface β_2_-AR expression and consequently insensitivity to the inhibitory effects of β_2_-AR agonists. These findings, in conjunction with our previous findings showing corticosteroid insensitivity in severe asthma fibrocytes, highlight a possible mechanism underlining the accumulation of fibrocytes in the circulation and lung tissue of severe asthma patients [6, 8]. Moreover, we show that combination of β_2_-AR agonists with corticosteroids are more effective in suppressing fibrocyte number and differentiation than each drug alone highlighting the importance of combination therapy in the treatment of remodelling in severe asthma.

## Additional files

Additional file 1: Supplementary Materials and Methods. (DOCX 19 kb)

Additional file 2: Figure S3. Number of fibrocytes and differentiating fibrocytes in NANT cells after culture in the absence or presence of salmeterol. The number of fibrocytes (% Col I^+^/CD45^+^; A) and differentiating fibrocytes (% α-SMA^+^; B) within the NANT cell population, were determined in the NANT cells from healthy subjects (*n* = 8–9) and patients with non-severe (*n* = 7) or severe asthma (n = 7–9) at day 0 (0 d) or after 3 days (3 d) in the absence or presence of salmeterol (10^−9^- 10^−7^ M). Data points represent mean ± SEM. * *p* < 0.05 and ** *p* < 0.01 versus vehicle-treated cells for each group. ^###^ *p* < 0.001 versus healthy group and ^$$$^ *p <* 0.001 versus non-severe asthma group. (PDF 478 kb)

Additional file 3: Figure S1. α–smooth muscle actin-positive cells also express both collagen I and CD45. NANT cells from one healthy subject were harvested after 3 days in culture and stained with antibodies for collagen I (Col I), α- smooth muscle actin (SMA) and CD45 or their respective IgG isotype controls. (**A** and **C**). The percentage of Col I+/α-SMA+ (**B**) and CD45+/ α-SMA+ cells (**D**) was determined by flow cytometry. (PDF 112 kb)

Additional file 4: Figure S2. Effect of salmeterol on NANT cell apoptosis. NANT cells from healthy subjects (*n* = 6) were treated with salmeterol (10^−8^ M) for 3 days and the percentage of live, and early and late apoptotic NANT cells was determined. Bars represent mean ± SEM. (PDF 173 kb)

Additional file 5: Figure S4. Effect of ICI-118,551 on salmeterol-mediated reduction in fibrocyte number and differentiation. NANT cells from healthy subjects (n = 6) were treated with salmeterol (10^−8^ M) in the presence or absence of ICI-118,551 (10^−5^ M) for 3 days. The number of fibrocytes (Col I^+^/CD45^+^ cells; A) and differentiating fibrocytes (α-SMA^+^ cells; B) was determined. Bars represent mean ± SEM.* *p* < 0.05 and ** *p* < 0.01. (PDF 69 kb)

## Abbreviations

ASM: Airway smooth muscle
GR: Glucocorticoid receptor
MR: Muscarinic receptor
NANT: Non-adherent non-T cell
PBMCs: Peripheral blood mononuclear cells
α-SMA: α-smooth muscle actin
β_2_-AR: β_2_-adrenergic receptor
