# Supplementary material for: Reduced suppressive effect of β2-adrenoceptor agonist on fibrocyte function in severe asthma
Source: Respir Res. 2017 Nov 21;18:194. doi: 10.1186/s12931-017-0678-7 (PMC5697384; doi:10.1186/s12931-017-0678-7)
Supplement: Supplementary file 1 — Supplementary Materials and Methods. (DOCX 19 kb) [file 12931_2017_678_MOESM1_ESM.docx]

***ONLINE DATA SUPPLEMENT***

Reduced suppressive effect of β_2_-adrenoceptor agonist on fibrocyte function

in severe asthma

Chun-Yu Lo MD, Charalambos Michaeloudes, Pankaj K Bhavsar, Chien-Da Huang, Po-Jui Chang, Chun-Hua Wang, Han-Pin Kuo and Kian Fan Chung

## MATERIALS AND METHODS

**Materials**

Salmeterol, dexamethasone, rolipram and ICI-118,551 hydrochloride were purchased from Sigma-Aldrich (Ayrshire, UK). Tiotropium bromide was purchased from Biorbyt Ltd (Cambridge, UK) and 8-Br-cAMP from Abcam (Cambridge, UK).

**Subjects**

Patients with severe and non-severe asthma and healthy subjects were recruited as previously described (Table 1).^1^ Patients with asthma had FEV_1_ reversibility of less than 12% or provocative concentration of methacholine causing a 20% fall in FEV_1_ (PC20) of less than 8 mg/mL. Patients with severe asthma were selected according to the American Thoracic Society guidelines for refractory asthma.^2^ They had at least one of two major criteria for corticosteroid usage, and at least two minor criteria of ongoing asthma. Patients who did not meet these criteria were included in the non-severe asthma group. Smokers and ex-smokers with a smoking history of greater than 5 pack-years and patients with co-morbidities were excluded. Informed consent was obtained from each participant. The study was approved by the Royal Brompton & Harefield NHS Trust/National Heart & Lung Institute Ethics Committee (London - Chelsea REC 08/H0708/109).

Cell apoptosis (shown in supplementary) was determined by FITC Annexin V/Dead Cell Apoptosis Kit with FITC annexin V and propidium iodide (Invitrogen –Life Technologies, Paisley, UK , V13242) using flow cytometry. Cell proliferation (shown in supplementary) was determined by Click-iT® 5-ethynyl-2'-deoxyuridine (EdU) Flow Cytometry Assay Kit with Pacific Blue™ azide (Invitrogen –Life Technologies, C10636) using flow cytometry.

**REFERENCES**

E1. Lo, C. Y., C. Michaeloudes, P. K. Bhavsar, C. D. Huang, C. H. Wang, H. P. Kuo, and K. F. Chung. 2015. Increased phenotypic differentiation and reduced corticosteroid sensitivity of fibrocytes in severe asthma. *J Allergy Clin.Immunol.* 135:1186-1195.

E2. 2000. Proceedings of the ATS workshop on refractory asthma: current understanding, recommendations, and unanswered questions. American Thoracic Society. *Am J Respir.Crit Care Med.* 162:2341-2351.
