# Supplementary figures and images for: Reduced suppressive effect of β2-adrenoceptor agonist on fibrocyte function in severe asthma
Source: Respir Res. 2017 Nov 21;18:194. doi: 10.1186/s12931-017-0678-7 (PMC5697384; doi:10.1186/s12931-017-0678-7)

Supplementary Figure S3

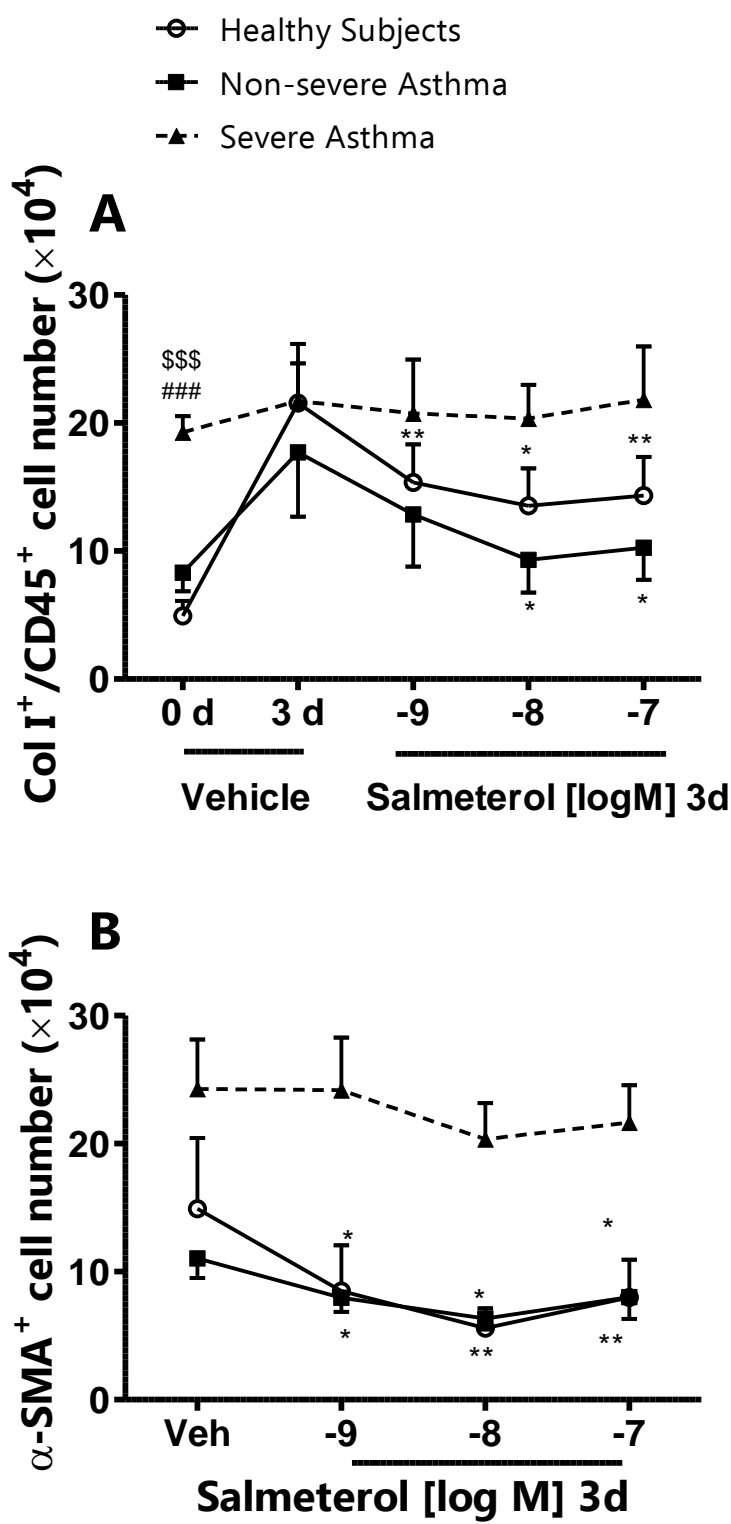

Supplement: Supplementary file 2 — Number of fibrocytes and differentiating fibrocytes in NANT cells after culture in the absence or presence of salmeterol. The number of fibrocytes (% Col I+/CD45+; A) and differentiating fibrocytes (% α-SMA+; B) within the NANT cell population, were determined in the NANT cells from healthy subjects (n = 8–9) and patients with non-severe (n = 7) or severe asthma (n = 7–9) at day 0 (0 d) or after 3 days (3 d) in the absence or presence of salmeterol (10−9- 10−7 M). Data points represent mean ± SEM. * p < 0.05 and ** p < 0.01 versus vehicle-treated cells for each group. ### p < 0.001 versus healthy group and $$$ p < 0.001 versus non-severe asthma group. (PDF 478 kb) [file 12931_2017_678_MOESM2_ESM.pdf]
