# Supplementary material for: Reduced suppressive effect of β2-adrenoceptor agonist on fibrocyte function in severe asthma
Source: Respir Res. 2017 Nov 21;18:194. doi: 10.1186/s12931-017-0678-7 (PMC5697384; doi:10.1186/s12931-017-0678-7)
Supplement: Supplementary file 3 — α–smooth muscle actin-positive cells also express both collagen I and CD45. NANT cells from one healthy subject were harvested after 3 days in culture and stained with antibodies for collagen I (Col I), α- smooth muscle actin (SMA) and CD45 or their respective IgG isotype controls. (A and C). The percentage of Col I+/α-SMA+ (B) and CD45+/ α-SMA+ cells (D) was determined by flow cytometry. (PDF 112 kb) [file 12931_2017_678_MOESM3_ESM.pdf]

Supplementary Figure S1

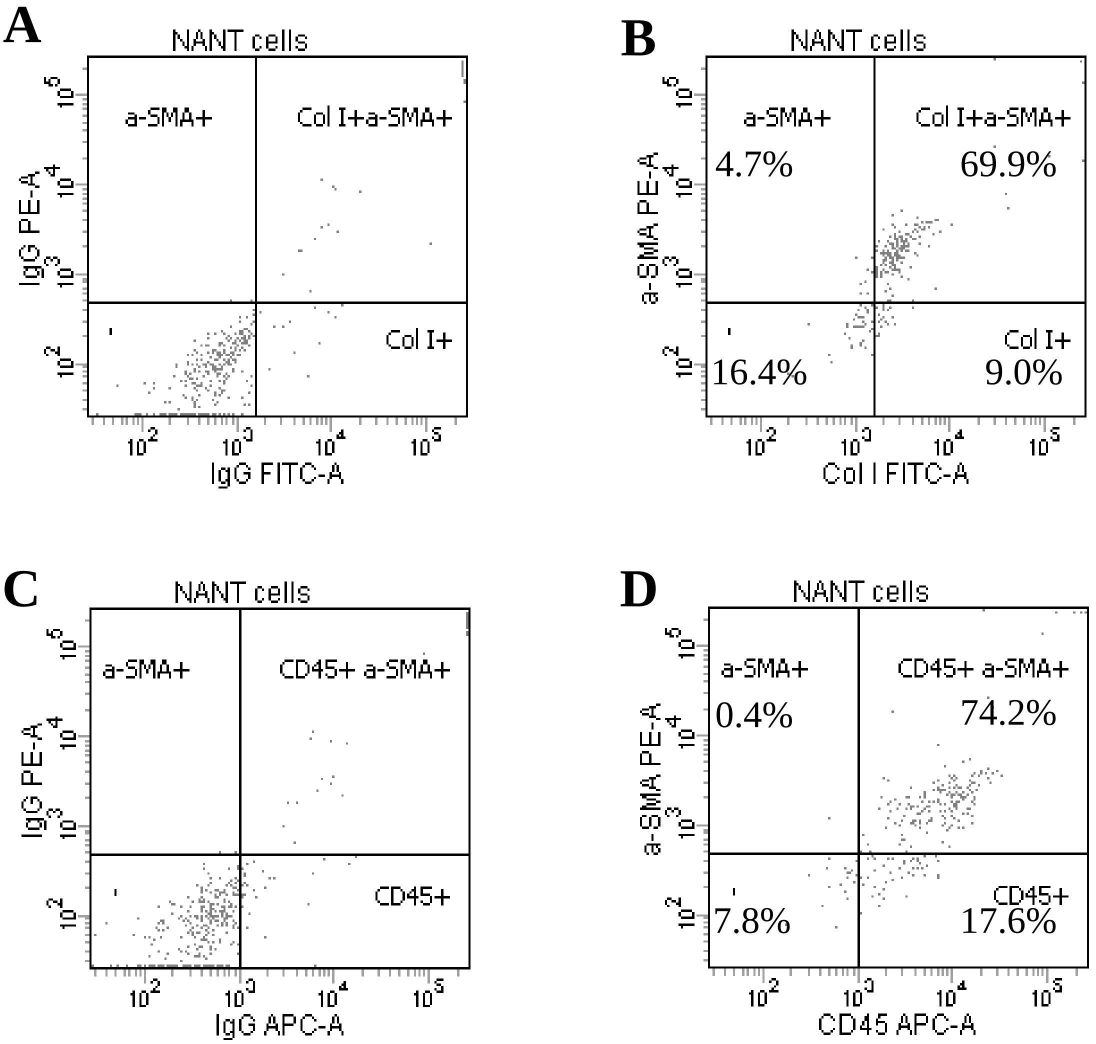

Figure S1: ***The expression of collagen I and CD45 on α–smooth muscle actin-positive cells.*** NANT cells from one healthy subject were harvested after 3 days in culture and stained with antibodies for collagen I (Col I), α- smooth muscle actin (SMA) and CD45 or their respective IgG isotype controls (**A** and **C**). The percentage of Col I+/α-SMA+ (**B**) and CD45+/ α-SMA+ cells (**D**) was determined by flow cytometry.
