# Supplementary material for: Reduced suppressive effect of β2-adrenoceptor agonist on fibrocyte function in severe asthma
Source: Respir Res. 2017 Nov 21;18:194. doi: 10.1186/s12931-017-0678-7 (PMC5697384; doi:10.1186/s12931-017-0678-7)
Supplement: Supplementary file 4 — Effect of salmeterol on NANT cell apoptosis. NANT cells from healthy subjects (n = 6) were treated with salmeterol (10−8 M) for 3 days and the percentage of live, and early and late apoptotic NANT cells was determined. Bars represent mean ± SEM. (PDF 173 kb) [file 12931_2017_678_MOESM4_ESM.pdf]

Supplementary Figure S2

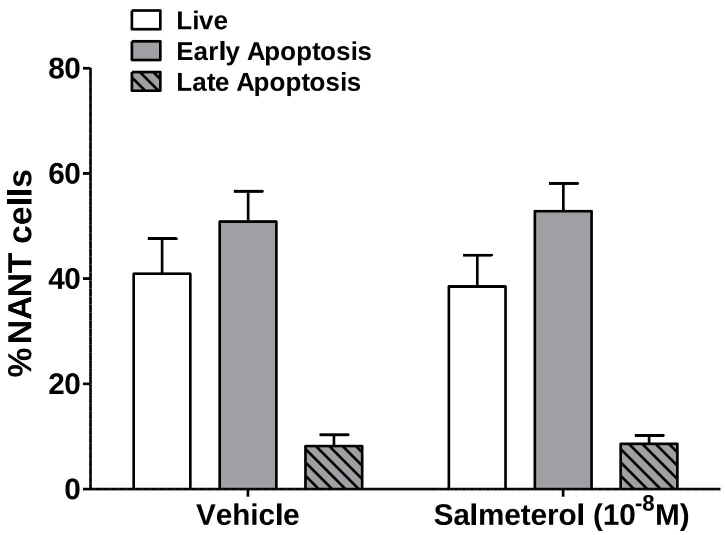

Figure S2: Effect of salmeterol on NANT cell apoptosis. NANT cells from healthy subjects (n=6) were treated with salmeterol (10^{-8}M) for 3 days and the percentage of live, and early and late apoptotic NANT cells was determined. Bars represent mean ± SEM.
