# Supplementary material for: Reduced suppressive effect of β2-adrenoceptor agonist on fibrocyte function in severe asthma
Source: Respir Res. 2017 Nov 21;18:194. doi: 10.1186/s12931-017-0678-7 (PMC5697384; doi:10.1186/s12931-017-0678-7)
Supplement: Supplementary file 5 — Effect of ICI-118,551 on salmeterol-mediated reduction in fibrocyte number and differentiation. NANT cells from healthy subjects (n = 6) were treated with salmeterol (10−8 M) in the presence or absence of ICI-118,551 (10−5 M) for 3 days. The number of fibrocytes (Col I+/CD45+ cells; A) and differentiating fibrocytes (α-SMA+ cells; B) was determined. Bars represent mean ± SEM.* p < 0.05 and ** p < 0.01. (PDF 69 kb) [file 12931_2017_678_MOESM5_ESM.pdf]

Supplementary Figure S4

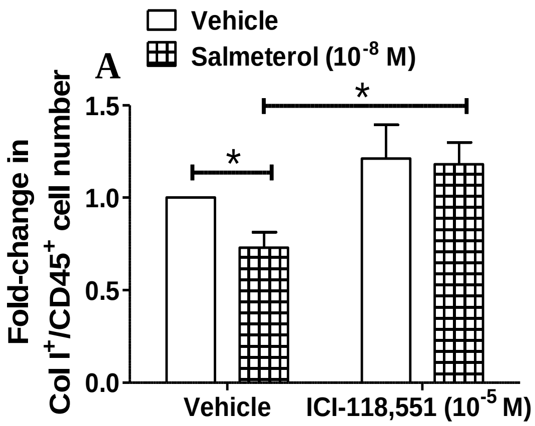

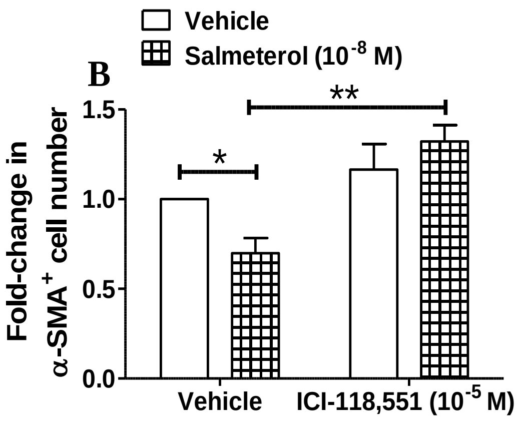

<u>Figure S4:</u> Effect of ICI-118,551 on salmeterol-mediated reduction in fibrocyte number and differentiation. NANT cells from healthy subjects (n=6) were treated with salmeterol (10^{-8}M) in the presence or absence of ICI-118,551 (10^{-5}M) for 3 days. The number of fibrocytes (Col I^+/$CD45^+$ cells; A) and differentiating fibrocytes (α-SMA^+ cells; B) was determined. Bars represent mean ± SEM.* $p < 0.05$ and ** $p < 0.01$.
